# Targeted therapy in pulmonary veno-occlusive disease: time for a rethink?

**DOI:** 10.1186/s12890-019-1031-3

**Published:** 2019-12-19

**Authors:** Qin Luo, Qi Jin, Zhihui Zhao, Qing Zhao, Xue Yu, Lu Yan, Yi Zhang, Changming Xiong, Zhihong Liu

**Affiliations:** Center for Pulmonary Vascular Diseases, Fuwai Hospital, National Center for Cardiovascular Diseases, Chinese Academy of Medical Sciences and Peking Union Medical College, 167 Beilishi Road, Xicheng District, Beijing, 100037 China

**Keywords:** Pulmonary veno-occlusive disease, Pulmonary arterial hypertension, Targeted therapy

## Abstract

**Background:**

Pulmonary veno-occlusive disease (PVOD) is a rare condition with poor prognosis, and lung transplantation is recommended as the only curative therapy. The role of pulmonary arterial hypertension targeted therapy in PVOD remains controversial, and long-term effects of targeted therapy have been rarely reported. This study aims to retrospectively evaluate the role of targeted therapy in PVOD patients and the long-term outcome.

**Methods:**

PVOD patients with good responses to targeted therapies were analyzed, and data pre- and post- targeted therapies were compared. An overview of the effects of targeted therapies on PVOD patients was also conducted.

**Results:**

Five genetically or histologically confirmed PVOD patients received targeted therapies and showed good responses. Their mean pulmonary arterial pressure by right heart catheterization was 62.0 ± 11.7 mmHg. Two receiving monotherapy got stabilized, and three receiving sequential combination therapy got improved, cardiac function and exercise capacity significantly improved after treatments. No pulmonary edema occurred. The mean time from the first targeted therapy to the last follow up was 39.3 months, and the longest was 9 years. A systematic review regarding the effects of targeted therapies on PVOD patients indicated majorities of patients got hemodynamics or 6-min walk distance improved, and 26.7% patients developed pulmonary edema. The interval from targeted drugs use to death ranged from 71 min to over 4 years.

**Conclusions:**

Cautious use of targeted therapy could safely and effectively improve or stabilize hemodynamics and exercise capacity of some patients without any complications. PVOD patients could live longer than expected.

## Background

Pulmonary veno-occlusive disease (PVOD), a subgroup of pulmonary arterial hypertension (PAH), is a rare and devastating condition histologically characterized by occlusion of pulmonary veins and venules with widespread fibrous intimal thickening and patchy capillary proliferation. Although PVOD shares similar clinical features with idiopathic PAH (IPAH), it progresses more rapidly even while on therapy and carries a worse prognosis with a dismal 1-year mortality rate of 72% [1].

Targeted therapies involving prostacyclin, endothelin-1 and nitric oxide signaling pathways have remarkably improved the prognosis of PAH patients, nevertheless, their roles in PVOD remain controversial for life-threatening drug-induced pulmonary edema. Only a few studies reported the effectiveness of vasodilators in PVOD without severe complications [2, 3]. It is currently recommended once PVOD is suspected, lung transplantation, the only curative therapy, should be considered as soon as possible [4]. In this study, we retrospectively analyzed five genetically or histologically confirmed PVOD patients with good responses to PAH-targeted drugs and no pulmonary edema, and shared our experience in the management and treatment of PVOD.

## Methods

### Patients

We retrospectively reviewed clinical data and outcomes from five PVOD patients hospitalized in the Center for Pulmonary Vascular Diseases (Fuwai Hospital, National Center for Cardiovascular Diseases, Chinese Academy of Medical Sciences and Peking Union Medical College, Beijing) between May 2009 and March 2018. PVOD was histologically confirmed by extensive involvement of pulmonary veins and venules with fibrous thickening of the intima on lung explants obtained after lung transplantation, or was clinically diagnosed if all following criteria were met: precapillary pulmonary hypertension by right heart catheterization (RHC), presence of two or more radiological characteristics of PVOD on chest high-resolution computed tomography (HRCT) (interlobular septal thickening, centrilobular ground-glass opacities, and mediastinal lymph node enlargement), low diffusing capacity for carbon monoxide, and mutations of the eukaryotic translation initiation factor 2 alpha kinase 4 (*EIF2AK4*) gene [5]. IPAH was defined by a mean pulmonary arterial pressure (mPAP) ≥25 mmHg at rest with pulmonary capillary wedge pressure ≤ 15 mmHg and pulmonary vascular resistance (PVR) > 3 Wood units, without known causes (connective tissue diseases, HIV infection, portal hypertension, congenital heart diseases and schistosomiasis), as we previously mentioned [6–8]. Good response to PAH targeted therapy was defined as improvement in New York Heart Association Functional Class (NYHA FC) with increased 6-min walk distance (6MWD), or stable condition without any deterioration of NYHA FC and 6MWD within 6 months. Signed written informed consents were obtained from all patients.

### Hemodynamic measurements

Transthoracic doppler echocardiography was routinely performed on admission and at every follow-up, right ventricular end-diastolic diameter (RVED), left ventricular end-diastolic diameter (LVED), tricuspid annular plane systolic excursion (TAPSE), systolic pulmonary artery pressure (sPAP), left ventricular ejection fraction (LVEF) and pericardial effusion were documented, sPAP was estimated by tricuspid regurgitation velocity (TRV) and right atrium pressure (RAP), sPAP = 4TRV^2^ + RAP. Hemodynamic evaluation by RHC was performed at baseline in all patients as we previously reported [6, 9]. RHC was performed by experienced physicians during hospitalization, and hemodynamic data including mixed venous oxygen saturation (SvO_2_), mean RAP (mRAP), right ventricular systolic pressure (RVSP), sPAP, mPAP, total pulmonary resistance (TPR), cardiac output (CO) and cardiac index (CI) were recorded.

### Clinical and functional assessment

Baseline data including sex, age, body mass index (BMI), symptom and signs, and medical history after initial admission were recorded. HRCT and pulmonary function tests were blindly performed and analyzed by experienced radiologists and medical technologists, and aforementioned radiological features, diffusing lung capacity of carbon monoxide (expressed as DLCO % pred), and DLCO/alveolar volume (expressed as DLCO/VA % pred) were recorded. NYHA FC, cardiothoracic ratio by chest X-ray, hemodynamic data by echocardiography and RHC, arterial blood gases, complete blood counts, renal and liver function tests, N-terminal prohormone brain natriuretic peptide (NT-proBNP), and cardiopulmonary exercise testing measurements pre- and post- PAH-targeted therapies were detailly reviewed. Data after targeted therapies were obtained when patients achieved the best values for LVEF by echocardiography or cardiac index by RHC [10]. Screening for *EIF2AK4* mutations was performed by Novogene Corporation, Beijing, China. Time from the first use of targeted therapy to the last follow up (April 2019) was described as survival time.

### Medical therapies

Dosage and administration of anticoagulants, diuretics, digoxin and PAH-targeted drugs including endothelin receptor antagonists (ERAs: bosentan, ambrisentan, macitentan), phosphodiesterase type 5 inhibitors (PDE5is: sildenafil, tadalafil), the soluble guanylate cyclase stimulator (riociguat), prostanoids (beraprost, iloprost, epoprostenol, treprostinil), and selective prostacyclin receptor agonist (selexipag) were detailly reviewed. For PVOD patients in NYHA FC III/IV, the intravenous infusion rate of treprostinil was initiated at 1.25 ng/kg/min, and was adjusted in increments of 1.25–2.5 ng/kg/min per day later. If not tolerated, the infusion rate was gradually reduced.

### Systematic review

All studies or case reports in English regarding patients with PVOD receiving PAH-targeted drugs were selected. Inclusion criteria were as follows: a. Patients were genetically or histologically diagnosed with PVOD; b. Patients with PVOD received above PAH-targeted drugs; c. At least one of mPAP, PVR, CO, CI and 6MWD pre- and post- PAH-targeted therapies was documented, if not available, dosage of targeted drugs and outcomes (6MWD and survival time) had to be included. All selected articles were screened by two independent reviewers, any discordances were resolved by further discussion. Data including demographics and hemodynamic measurements by RHC (mPAP, PVR, CO and CI), drug name and dosage, outcomes and complications (6MWD, time from targeted drug use to death and pulmonary edema) were extracted.

### Statistical analysis

Statistical analysis was performed using SPSS version 23.0 (IBM Corp., Armonk, NY, USA). Results were presented as mean ± standard deviation for continuous variables. Differences pre- and post- PAH-targeted therapies were compared using paired t-test and drawn by GraphPad Prism Version 5.0 (GraphPad Software Inc., San Diego, CA, USA). A two-sided *P* < 0.05 was considered statistically significant.

## Results

### Baseline characteristics

**Fig. 1:**
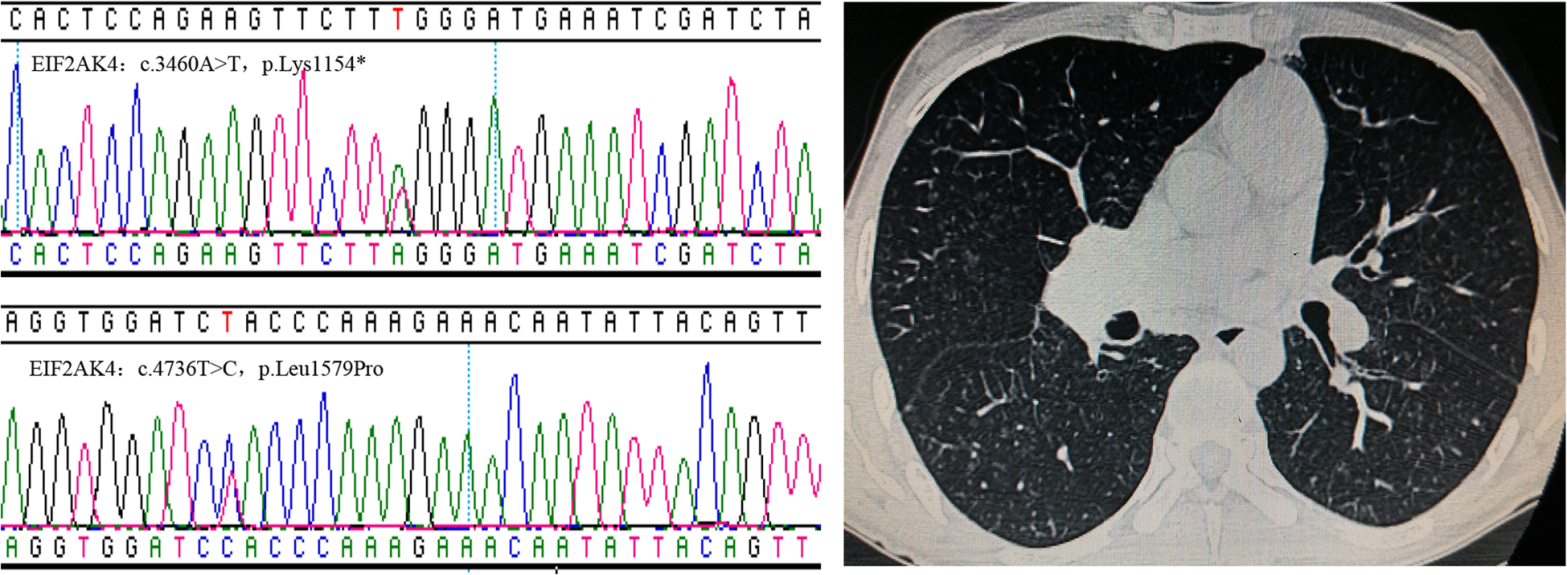
Representative images of PVOD patients. EIF2AK4 gene mutation sites (left) and chest computed tomography displaying interlobular septal thickening and ground glass opacities (right)

Between May 2009 and March 2018, five patients with PVOD (34.2 ± 5.0 years) responding well to targeted therapies in our center were collected (Table 1). All patients complained of chest tightness and/or shortness of breath, and only one patient presented with edema of lower extremities on admission which disappeared after diuretic therapy. At least two of characteristic HRCT features including interlobular septal thickening, ground glass opacities and enlarged mediastinal lymph nodes were found in each subject. Moreover, severe diffusion dysfunction with a decreased DLCO % pred (31.1 ± 2.9%) was observed. *EIF2AK4* gene mutation, a cause of heritable PVOD, was found in all cases (Fig. 1, Table 1). Patient 5 was misdiagnosed with IPAH and received PAH-targeted therapies for over 9 years, and he was confirmed as PVOD by biopsy of lung explants obtained after lung transplantation. Three patients were exposed to smoking, a potential risk factor strongly associated with PVOD [1]. Time from onset to admission ranged from 1 to 84 months.

**Table 1:** Basic characteristics of patients with PVOD

Patient number	1	2	3	4	5
Sex	M	F	M	M	M
Age ranges (year)	35–40	35–40	30–35	32–37	25–30
BMI (kg/m^2^)	18.0	18.7	21.9	22.4	15.5
Chest tightness / Shortness of breath	Yes	Yes	Yes	Yes	Yes
Edema of lower extremities	No	No	Yes	No	No
HRCT					
Interlobular septal thickening	Yes	Yes	No	Yes	Yes
Ground glass opacities	Yes	Yes	Yes	Yes	Yes
Enlarged mediastinal lymph nodes	No	No	Yes	No	No
EIF2AK4 mutation	c.2488C > T; c.989_990del	c.2965C > T; c.4724 T > C	c.1948C > T	c.3460A > T; c.4736 T > C	c.4414_4417del
Medical history					
Smoking	Yes	No	No	Yes	Yes
Drinking	No	No	No	Yes	No
Acute coronary disease	No	No	No	No	No
Hypertension	No	No	No	No	No
Hyperlipemia	No	No	No	No	No
Diabetes	No	No	No	No	No
Obstructive sleep apnea	No	No	No	No	No
Time from onset to admission (month)	72	1	84	60	6

*BMI* Body mass index, *HRCT* High resolution computed tomography, *EIF2AK4* Eukaryotic translation initiation factor 2 alpha kinase 4

### Clinical variables pre- and post- targeted therapies

**Fig. 2:**
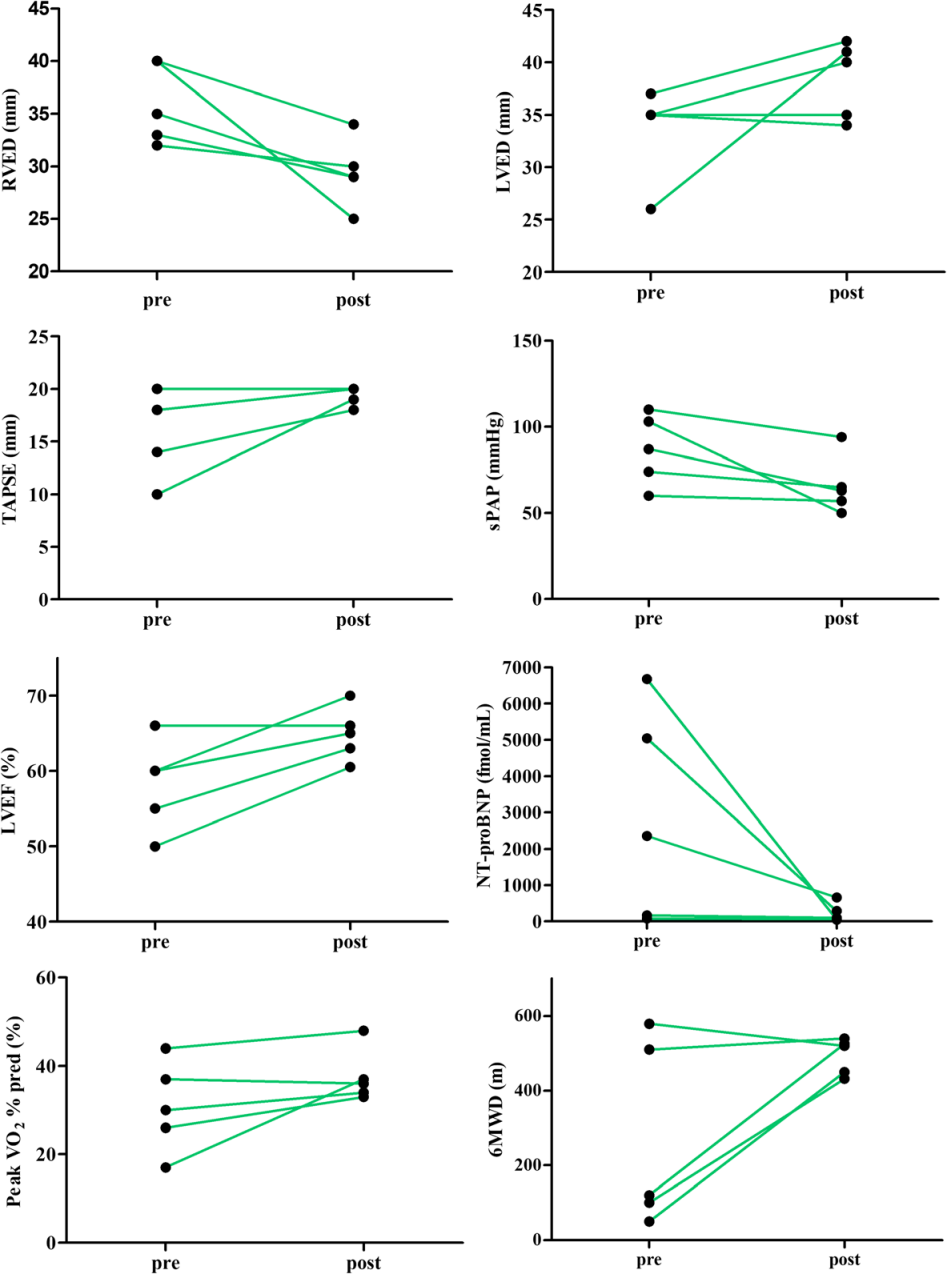
Changes of variables pre- and post- PAH targeted therapies

Clinical variables pre- and post- targeted therapies were shown in Additional file 1: Table S1. Three patients were in NYHA FC III/IV on admission and got improved to NYHA FC II after treatments. RVED decreased significantly compared to that on admission (36.0 ± 3.8 vs 29.4 ± 3.2 mm, *P* < 0.05). sPAP estimated by echocardiography and mPAP measured by RHC were 86.8 ± 20.5 mmHg and 62.0 ± 11.7 mmHg respectively. For some reasons such as economic issues, RHC was not performed in some patients post-targeted therapies. NT-proBNP and 6MWD were both remarkably improved in Patient 1/3/5 after treatments (Additional file 1: Table S1; Fig. 2). The mean time from the first use of targeted therapy to the last follow up was 39.3 months, four are still in good condition, the last was re-hospitalized several times for alcohol intoxication or volume overload during 9 years and underwent lung transplantation in May 2018, and histopathologic examination confirmed intimal remodeling of veins and venules.

### General treatment and PAH-targeted therapy

Table 2 summarizes the medical therapies for our patients. Four were anticoagulated with warfarin. All took diuretics, three were treated with at least two kinds of diuretics. All received targeted therapies, two were given monotherapy, two with sequential dual combination therapy (tadalafil or sildenafil combined with treprostinil), one with sequential quadruple combination therapy. All responded well to targeted therapies without pulmonary edema occurrence (See Additional file 1: Figure S1), no symptoms such as hemoptysis or pink frothy sputum occurred among our patients when given targeted drugs, NT-proBNP levels decreased, and their arterial oxygen saturation and DLCO got improved, some patients had no supplemental oxygen requirement and maintained oxygen saturation > 95% after treatments. Notable clinical and hemodynamic improvements with increased cardiac function and 6MWD were observed in three patients in NYHA FC III/IV, and two patients in NYHA FC II treated with tadalafil or sildenafil achieved stabilized condition for over 36 and 13.3 months respectively. Patient 5 received inhaled iloprost at his first onset in May 2009, and sildenafil was added on since July 2009. In June 2013, ambrisentan was sequentially combined, and treprostinil was added from September 2016 till his transplantation in May 2018.

**Table 2:** General treatment and PAH targeted therapy in patients with PVOD

Patient number	1	2	3	4	5
Warfarin	2.25 mg, po, qod	2.25 mg, po, qd	No	5 mg, po, qd	2 mg, po, qd
Diuretics					
Spironolactone	No	20 mg, po, qd	2No	20 mg, po, qd	20 mg, po, qd
Furosemide	40 mg, po, qod	No	20 mg, po, qod	No	20 mg, po, qod
Torsemide	20 mg, po, qod	No	10 mg, po, qod	No	10 mg, po, qod
Digoxin	0.125 mg, po, qd	No	0.25 mg, po, qd	No	0.125 mg, po, qd
Targeted therapies					
ERAs	No	No	No	No	Ambrisentan, 10 mg, po, qd
PDE5is	Tadalafil, 10 mg, po, qd	Tadalafil, 10 mg, po, qd	Sildenafil, 20 mg, po, tid	Sildenafil, 20 mg, po, tid	Sildenafil, 20 mg, po, tid
Prostanoids	Treprostinil, iv, 15.25 ng/kg/min	No	Treprostinil, iv, 17.5 ng/min/kg	No	Iloprost, 1 ml, inh, q3h; Treprostinil, iv, 20 ng/min/kg

*ERAs* Endothelin receptor antagonists, *PDE5is* Phosphodiesterase type 5 inhibitors

### Systematic review of patients with PVOD receiving PAH-targeted drugs

No prospective cohort studies or randomized controlled trials have been performed by now. Sixteen case reports and one retrospective study between 1989 and 2018 met the inclusion criteria (Additional file 1: Table S2) [2, 11–26]. A total of 30 patients with PVOD receiving PAH-targeted drugs during their hospitalizations were reported, and 16 out of them (53.3%) were male. Sixteen patients received monotherapy [2, 11–17, 19, 22], the commonest targeted drug used for these patients was epoprostenol (75%), and 12 patients were treated with sequential dual combination therapy [2, 18, 21, 24–26], one with initial dual combination therapy [2], one with sequential triple combination therapy, bosentan 125 mg bid stabilized his condition for 7 months, sildenafil 40 mg tid as an add-on therapy enabled remarkable clinical stabilization with a 6MWD of 560 m, then a low dose of epoprostenol (1–2 ng/kg/min) was added for declined exercise capacity but was stopped due to deterioration of oxygen saturation, sequential iloprost 2.5 μg q4h maintained his status till double lung transplantation [23]. Sildenafil was demonstrated to be effective and safe [17], and it is one of the most frequently used drugs in combined therapy for PVOD patients [18, 21, 23–26], those receiving sildenafil as monotherapy [17] or combined therapy [18, 21, 23, 25, 26] almost survived more than 1 year. Pulmonary edema following targeted drugs occurred in eight patients (26.7%) [2, 12, 13, 16, 19, 21–23], and epoprostenol seemed to induce pulmonary edema more frequently. Gratifyingly, some patients got hemodynamics or 6MWD improved more or less even though pulmonary edema happened [2, 12, 16, 19, 22, 23], and one of them even lived to 4 years after diagnosis [23]. The time from targeted drugs use to death ranged from 71 min to more than 4 years.

## Discussion

PVOD is a rare cause of pulmonary hypertension, and is currently classified as a subgroup of PAH. The exact prevalence of PVOD remains unknown, because many cases are probably misdiagnosed as IPAH due to similar clinical and hemodynamic features and lack of histological or genetic confirmation. It was estimated PVOD accounted for around 10% cases of IPAH [4]. Unlike IPAH which has a female predominance, PVOD seems to affect men and women equally [27]. Its molecular pathogenesis remains unclear, risk factors such as genetic factors, chemicals and chemotherapies, autoimmunity and inflammation, and cigarette smoking exposure have been reported to be associated with the development of PVOD [1, 5], and three of our patients got tobacco exposure which caused endothelial dysfunction and induced vascular remodeling in animal models of pulmonary hypertension.

The pathological feature of PVOD is characterized by diffuse intimal fibrosis of the small veins and venules. Histopathological examination remains the gold standard for a definitive diagnosis of PVOD, however, lung biopsy is contraindicated due to unacceptable high risk of bleeding. A combination of clinical findings, low diffusing capacity for carbon monoxide and characteristic signs on chest HRCT is recommended to diagnose PVOD [4]. All our patients presented with dyspnea, severe diffusion dysfunction with DLCO/ VA % pred < 55%, and decreased exercise capacity, and had at least two radiological signs of PVOD described on HRCT. *EIF2AK4* gene was identified as the major gene in the families with heritable PVOD in 2014 [28], and identification of a biallelic *EIF2AK4* mutation is practically enough to diagnose PVOD even without histological confirmation [4]. A recent study reported a patient carrying biallelic *EIF2AK4* gene mutation displayed good response to PAH-targeted therapy and was alive for over 3 years [26], similarly, all our patients were detected with biallelic *EIF2AK4* mutations and showed good responses, but the prognoses of patients with biallelic mutations in *EIF2AK4* varied [29], indicating possibly favorable effects of PAH-targeted drugs in patients harboring specific *EIF2AK4* gene mutations.

PVOD prognosis is worse than other subtypes of PAH with a 72% mortality within 1 year after diagnosis [15]. PAH-targeted therapy prominently improved the outcome of PAH patients, yet evidences on the use of PAH-targeted therapy in PVOD are still weak and conflicting, it is generally recognized that targeted therapy can be harmful and occasionally fatal in PVOD for severe complications, thus vasodilators are only recommended as a bridge to transplantation. However, our study conferred different perspectives, the mean time from targeted therapy to last follow up was 39.3 months, one patient (Patient 5) was previously misdiagnosed with IPAH and received sequential combination therapies, biallelic EIF2AK4 mutations were detected in June 2017, and PVOD was finally considered when combined with his clinical characteristics. However, lung transplantation was postponed due to his unwillingness till May 2018. To the best of our knowledge, the longest duration under monotherapy (beraprost) among PVOD patients was over 15 years after initial admission [30], and our patient had the longest 9-year duration under combined therapy. Additionally, some cases also reported over 2-year survival under targeted therapy [15, 16, 19, 23, 26], all of which forced us to rethink the role of PAH-targeted therapy in PVOD, we question is it really true that PVOD patients will deteriorate quickly and die within 1 year? Is lung transplantation still the only curative approach? Can targeted therapy only serve as a bridge to transplantation? Can we decide not to use targeted therapy just for fear of pulmonary edema? A recent systematic review regarding the efficacy and safety of vasodilators in PVOD patients showed vasodilators could effectively improve PVR, 6MWD and survival in some cases, but correlated with the risk of pulmonary edema in many cases [3]. Even pulmonary edema occurred, some patients could still tolerate and had startling prognoses for more than 2 years [16, 19, 23]. Long term clinical response to vasodilators is rarely reported, in our clinical investigation, PAH-targeted therapy can effectively improve cardiac function and 6MWD or long-termly stabilize the status at least for certain PVOD patients under large doses of diuretics without any pulmonary edema. Gene mutation screening may be considered, especially for those in their early stage, responses to targeted therapies at that time can be tested without leading to lethal pulmonary edema, Patient 2 and 4 are the best examples. Large doses of diuretics should be used when PAH-targeted therapies were prescribed. Anticoagulation is currently controversial among PVOD patients and should be individually administrated. Patient 1 was anticoagulated in consideration of his medical history of antiphospholipid syndrome and venous thromboembolism. Considering a relatively low risk of bleeding, Patients 2/4 received anticoagulation therapies before the results of gene detection came out. For Patient 3, he was admitted for the evaluation of lung transplantation in March 2018 and was genetically confirmed with PVOD, thus anticoagulation therapy was not prescribed. Patient 5 was previously misdiagnosed with IPAH, and therefore received anticoagulants for a long period.

Bosentan, an endothelin receptor antagonist, was reported to be effective in improving hemodynamics [20, 22, 27], however, pulmonary edema occurred in some cases and the outcomes varied [21–23]. Macitentan was first proved to be beneficial for PVOD in 2016, and brought a clear improvement when combined with sildenafil [25]. Here in our study, we for the first time reported the good effects of tadalafil combined with or without treprostinil on PVOD (Patient 1/2), and also presented the second successful example of ambrisentan in treating PVOD (Patient 5). Sildenafil, a specific inhibitor of PDE5, may have a better safety profile due to its mild impact on pulmonary wedge pressure, chronic administration of sildenafil was effective in enhancing exercise capacity of PVOD patient [17], for our patients, we usually started with sildenafil or tadalafil, ambrisentan was sequentially added on if no obvious effects were observed. It was previously revealed pulmonary capillary pressure increased with low-dose prostacyclin but decreased with higher doses [12], indicating low-dose prostacyclin might cause pulmonary edema. A recent study demonstrated cautious application of epoprostenol at a high dose (24.1 ± 9.4 ng/min/kg) improved exercise capacity and increased cardiac index, and worked successfully as a bridge to lung transplantation [10]. Before using treprostinil for severe patients, intravenous iloprost for its short half-life period was firstly attempted in our clinical practice. Careful titration of treprostinil which was initiated at 1.25 ng/kg/min, and then adjusted in rapid increments of 1.25–2.5 ng/kg/min per day later with necessary diuretics or inotropes, was proved to be safe and useful for our patients. Effectiveness of these targeted drugs on PVOD may be explained by vasodilating post-capillary vessels with higher doses. Initial dual combination therapy should be avoided due to highly possible pulmonary edema. Larger-scale studies with long-term follow-up should be carried out to confirm these findings.

Our study had several limitations. Five patients were included in this retrospective study, the sample size was relatively small. Besides, RHC was not conducted in some patients for safety and economic issues during follow-up, which could not best reflect the hemodynamic improvements under PAH-targeted therapy. Last but not the least, there is definitely a publication bias in PVOD literature since cases with good outcomes under targeted therapy have a higher probability of being published than those with dismal prognoses, moreover, we excluded studies with limited data in our systematic review, thus inevitably producing selection bias.

## Conclusions

We applied PAH-targeted therapies to 5 PVOD patients and achieved good responses without any severe complications. Cautious application of PAH-targeted therapy could safely and effectively improve or stabilize hemodynamics and exercise capacity of some patients without pulmonary edema. PVOD patients could live longer than expected.

## Supplementary information

**Additional file 1: Table S1.** Clinical variables pre- and post- PAH targeted therapies. **Table S2.** Systematic review of patients with PVOD receiving PAH targeted drugs. **Figure S1.** Chest X-ray and computed tomography pre- (A, C) and post- (B, D) PAH targeted therapy in Patient 3.

## Data Availability

All data generated or analyzed during this study are included in this manuscript.

## Abbreviations

6MWD: 6-min walk distance
BMI: Body mass index
CI: Cardiac index
CO: Cardiac output
DLCO/VA: Diffusing lung capacity of carbon monoxide/alveolar volume
EIF2AK4: Eukaryotic translation initiation factor 2 alpha kinase 4
ERAs: Endothelin receptor antagonists
HRCT: High resolution computed tomography
IPAH: Idiopathic PAH
LVED: Left ventricular end-diastolic diameter
LVEF: Left ventricular ejection fraction
mPAP: Mean pulmonary arterial pressure
mRAP: Mean RAP
NT-proBNP: N-terminal prohormone brain natriuretic peptide
NYHA FC: New York Heart Association Functional Class
PAH: Pulmonary arterial hypertension
PDE5is: Phosphodiesterase type 5 inhibitors
PVOD: Pulmonary veno-occlusive disease
PVR: Pulmonary vascular resistance
RAP: Right atrium pressure
RHC: Right heart catheterization
RVED: Right ventricular end-diastolic diameter
RVSP: Right ventricular systolic pressure
sPAP: Systolic pulmonary artery pressure
SvO_2_: Mixed venous oxygen saturation
TAPSE: Tricuspid annular plane systolic excursion
TPR: Total pulmonary resistance
TRV: Tricuspid regurgitation velocity
