# Supplementary material for: Targeted therapy in pulmonary veno-occlusive disease: time for a rethink?
Source: BMC Pulm Med. 2019 Dec 19;19:257. doi: 10.1186/s12890-019-1031-3 (PMC6924010; doi:10.1186/s12890-019-1031-3)
Supplement: Supplementary file 1 — Additional file 1: Table S1. Clinical variables pre- and post- PAH targeted therapies. Table S2. Systematic review of patients with PVOD receiving PAH targeted drugs. Figure S1. Chest X-ray and computed tomography pre- (A, C) and post- (B, D) PAH targeted therapy in Patient 3. [file 12890_2019_1031_MOESM1_ESM.docx]

Additional file 1: **Table S1** Clinical variables pre- and post- PAH targeted therapies

| Patient number | 1 | | 2 | | 3 | | 4 | | 5 | |
| --- | --- | --- | --- | --- | --- | --- | --- | --- | --- | --- |
| Treatment | Pre- | Post- | Pre- | Post- | Pre- | Post- | Pre- | Post- | Pre- | Post- |
| NYHA FC | III | II | II | II | IV | II | II | II | IV | II |
| Cardiothoracic ratio | 0.55 | 0.42 | 0.4 | 0.39 | 0.52 | 0.4 | 0.39 | 0.4 | 0.32 | 0.4 |
| Echocardiography |  |  |  |  |  |  |  |  |  |  |
| RVED (mm) | 40 | 34 | 32 | 30 | 40 | 25 | 33 | 29 | 35 | 29 |
| LVED (mm) | 35 | 34 | 35 | 35 | 26 | 41 | 37 | 42 | 35 | 40 |
| TAPSE (mm) | 14 | 18 | 18 | 20 | 10 | 19 | 20 | 20 | Normal | Normal |
| sPAP (mmHg) | 87 | 63 | 110 | 94 | 103 | 50 | 60 | 57 | 74 | 65 |
| LVEF (%) | 55 | 63 | 60 | 70 | 66 | 66 | 60 | 65 | 50 | 60.5 |
| Pericardial effusion | No | No | No | No | Yes | No | No | No | No | No |
| Right heart catheterization |  |  |  |  |  |  |  |  |  |  |
| SvO_2_ (%) | 69.6 | NM | 79.65 | NM | 63.9 | NM | 77.5 | NM | 61 | 71.9 |
| mRAP (mmHg) | 0 |  | 7 |  | 8 |  | 3 |  | 5 | 13 |
| RVSP (mmHg) | 91 |  | 85 |  | 124 |  | 86 |  | 81 | 64 |
| sPAP (mmHg) | 87 |  | 88 |  | 120 |  | 86 |  | 78 | 67 |
| mPAP (mmHg) | 62 |  | 58 |  | 82 |  | 55 |  | 53 | 49 |
| TPR (dyn•s•cm^-5^) | 906.06 |  | 759.38 |  | 1348.65 |  | 783.17 |  | 1380.53 | 821.83 |
| CO (L/min) | 5.69 |  | 8.4 |  | 4.56 |  | 6.93 |  | 3.07 | 4.55 |
| CI (L/min/m^2^) | 3.51 |  | 5.19 |  | 2.64 |  | 3.96 |  | 1.92 | 3.02 |
| Blood gas analysis |  |  |  |  |  |  |  |  |  |  |
| pH | 7.45 | 7.44 | 7.47 | 7.44 | 7.46 | 7.41 | 7.45 | 7.44 | 7.483 | 7.436 |
| pCO_2_ (mmHg) | 33 | 33.5 | 33 | 34 | 33 | 36.5 | 34 | 30.7 | 24.2 | 35.7 |
| pO_2_ (mmHg) | 51 | 56.9 | 65 | 63 | 60 | 80.7 | 56 | 69.1 | 79.4 | 93.1 |
| SaO_2_ (%) | 88 | 89.2 | 94 | 93 | 92 | 95.7 | 90 | 93.3 | 96.7 | 97 |
| Hematology |  |  |  |  |  |  |  |  |  |  |
| RBC (10^12^/L) | 6.47 | 7.41 | 5.24 | 5.66 | 5.38 | 5.08 | 5.65 | 5.83 | 6.04 | 5.72 |
| Hemoglobin (g/L) | 215 | 228 | 170 | 179 | 179 | 160 | 177 | 186 | 188 | 156 |
| RBC volume distribution width (%) | 15.7 | 16.9 | 12.5 | 13.5 | 13.6 | 13.3 | 13.4 | 13.4 | 14.2 | 12.6 |
| Biochemical tests |  |  |  |  |  |  |  |  |  |  |
| Total bilirubin (umol/L) | 45.33 | 26.2 | 24.28 | 13.2 | 71.7 | 19.6 | 24.3 | 23.8 | 31.6 | 18.3 |
| Direct bilirubin (umol/L) | 7.23 | 11.7 | 3.72 | 1.7 | 31.5 | 2.7 | 6 | 7.5 | 5.3 | 3.91 |
| Creatinine (umol/L) | 92.41 | 98.18 | 72.55 | 86.49 | 82.99 | 80.52 | 92.32 | 99.78 | 120 | 95.28 |
| Blood urea nitrogen (mmol/L) | 6.6 | 6.3 | 5.6 | 4.3 | 5.1 | 4.5 | 3.2 | 4.2 | 8.1 | 8.4 |
| Serum uric acid (umol/L) | 556.92 | 405.81 | 347.05 | 302.68 | 476.71 | 330.54 | 338.6 | 462.98 | 621.6 | 525.02 |
| NT-proBNP (fmol/mL) | 2353 | 663.3 | 164.1 | 97.3 | 6674 | 45.5 | 76.1 | 66.6 | 5044 | 283.3 |
| CPET |  |  |  |  |  |  |  |  |  |  |
| Peak VO_2_/kg (ml/min/kg) | 13.44 | 14.2 | 13.45 | 13.8 | 7.2 | 15.2 | 14.8 | 14.8 | 14.7 | 17.57 |
| Peak VO_2_ % pred (%) | 30 | 34 | 44 | 48 | 17 | 37 | 37 | 36 | 26 | 33 |
| Pulmonary Function Tests |  |  |  |  |  |  |  |  |  |  |
| DLCO % pred (%) | 32 | 36 | 32 | 41 | 26 | 36 | 33.5 | 29 | 32 | NA |
| DLCO/VA % pred (%) | 30 | 30 | 30 | 42 | 28 | 41 | 33 | 33 | 28.6 | NA |
| 6MWD (m) | 100 | 432 | 510 | 540 | 120 | 526 | 580 | 520 | 50 | 450 |
| Survival time (month) | 26 | | 36 | | 12.5 | | 13.3 | | 108.5 | |

*NYHA* New York Heart Association Functional Class*, RVED* Right ventricular end-diastolic diameter, *LVED* Left ventricular end-diastolic diameter, *TAPSE* Tricuspid annular plane systolic excursion, *sPAP* Systolic pulmonary artery pressure, *SvO_2_* Mixed venous oxygen saturation, mRAP Mean right atrium pressure, *LVEF* Left ventricular ejection fraction, *RVSP* Right ventricular systolic pressure, *mPAP* Mean pulmonary arterial pressure, *TPR* Total pulmonary resistance, *CO* Cardiac output, *CI* Cardiac index, *pCO_2_* Partial pressure of carbon dioxide, *pO2* Partial pressure of oxygen, *SaO_2_* Arterial oxygen saturation, *RBC* Red blood cell, *NT-proBNP* N-terminal prohormone brain natriuretic peptide, *CPET* Cardiopulmonary exercise testing, *DLCO* Diffusing lung capacity of carbon monoxide, *VA* Alveolar volume, *6MWD* 6-minute walk distance, *NM* Not measured, *NA* Not available

Additional file 1: **Table S2** Systematic review of patients with PVOD receiving PAH targeted drugs

| Year | Sex | mPAP (mmHg) | | PVR (WU) | | CO (L/min) | | CI (L/min/m^2^) | | 6MWD (m) | | Pulmonary edema | Drug | Dose | Effect | Time from drug to death | Ref |
| --- | --- | --- | --- | --- | --- | --- | --- | --- | --- | --- | --- | --- | --- | --- | --- | --- | --- |
|  |  | Pre- | Post- | Pre- | Post- | Pre- | Post- | Pre- | Post- | Pre- | Post- |  |  |  |  |  |  |
| 1989 | F | 51 | 46 | 21.4 | 13.5 | － | | 1.5 | 2.4 | － | | No | epo | 8.3 ng/kg/min | Improved | － | 11 |
| 1995 | F | 47 | － | 18.3 | 8.0 | 2.9 | 4.6 | － | | | | Yes | epo | 12 ng/kg/min | Improved | 1 month | 12 |
| 1998 | F | 72 | － | 22.5 | － | 2.7 | － | 1.3 | － | | | Yes | epo | 2 ng/kg/min | Deteriorated | 71 min | 13 |
| 1999 | M | 48 | 47 | 8.9 | 7.0 | 4.5 | 5.6 | 2.3 | 2.9 | 200 | 320 | No | ilo | 15ug | Improved | 9 weeks | 14 |
| 2000 | M | 47 | 37 | 6.5 | － | 6.0 | － | | | | | No | epo | － | Deteriorated | 6 months |  |
|  | F | 53 | 35 | 10.7 | － | 4.0 | － | | | | | No | epo | 13 ng/kg/min | Improved | Alive >2 years | 15 |
|  | M | 63 | 55 | 19 | － | 2.8 | － | | | | | No | epo | － | Deteriorated | 3 days |  |
| 2002 | M | 70 | 36 | 15.1 | － | 4.0 | － | | | 90 | 450 | Yes | epo | 29 ng/kg/min | Improved | 2 years | 16 |
| 2005 | M | 62 | 48 | － | | EF 75% | | － | | 112 | 408 | No | sil | 75mg tid | Improved | Alive >1 year | 17 |
| 2006 | M | sPAP 140 | 28 (sPAP 49) | － | 4.5 | － | | | 2.9 | － | | No | epo+sil | 36 ng/kg/min; 25mg tid | Improved | 18 months | 18 |
| 2008 | F | 69 | － | 18.7 | － | 3.0 | － | | | | 346.0 | Yes | epo+imatinib | 22 ng/kg/min; 200 mg bid | Improved | 28 months (alive) | 19 |
| 2008 | F | 48 | － | 15.3 | － | 2.6 | － | － | | 375.0 | 500.0 | No | bos | 250 mg/day | Improved | Over 6 months | 20 |
| 2009 | M 7/12 | 58±8 | 53±9 | 28.4±8.4 | 17±5.2 | － | | 1.99±0.68 | 2.94±0.89 | 281±162 | 322±160 | 1/12, mild and reversible | 4 epo and 8 bos+epo | 13 (5–22) ng/kg/min | Improved at least 3 months | 145/201/303/75/252 days; 7 alive (1 LT list, 6 LT) |  |
|  |  |  |  |  |  |  |  |  |  |  |  |  |  |  |  |  | 2 |
|  |  |  |  |  |  |  |  |  |  |  |  |  |  |  |  |  |  |
| 2009 | F | 45 | 58 | 9.6 | 17 | － | | 3.5 | 3.17 | 363 | 338 | Yes | bos+sil | － | Deteriorated | Alive >1 year after LT | 21 |
| 2009 | F | 70 | 63 | 24 | 13.8 | 2.5 | 4 | 1.4 | 2.4 | 200 | 500 | Yes | bos | 125 mg tid | Improved | 1 year | 22 |
| 2013 | M | 53 | － | 10.7 | － | 4 | － | | | 225 | 560 | Yes | bos+sil+ilo | 125 mg bid+40 mg tid+2.5 μg q4h | Improved | 4 years (alive) | 23 |
| 2016 | M | >60 | － | 13.78 | － | | | 2.0 | － | Improved | | No | sil+bos | － | No significant improvement | Referred for LT | 24 |
| 2017 | F | 36 | 42 | 12 | 6.4 | － | | 1.9 | 3.3 | 144 | 325 | No | mac+sil | 20mg tid; 10 mg/d | Improved | Over 1 year | 25 |
| 2018 | M | 76 | － | 3.2 | － | 2.7 | － | | | | | No | amb+sil | 5mg po qd; 20mg po q8h | Improved | Over 3 years | 26 |

mPAP Mean pulmonary arterial pressure, *PVR* Pulmonary vascular resistance, *CO* Cardiac output, *CI* Cardiac index, *6MWD* 6-minute walk distance, *F* female, *M* male, *epo* Epoprostenol, *ilo* Iloprost, *EF* Ejection fraction, *sPAP* Systolic pulmonary artery pressure, *sil* Sildenafil, bos, Bosentan, *LT* Lung transplantation, *mac* Macitentan, *amb* Ambrisentan, *－*Not known


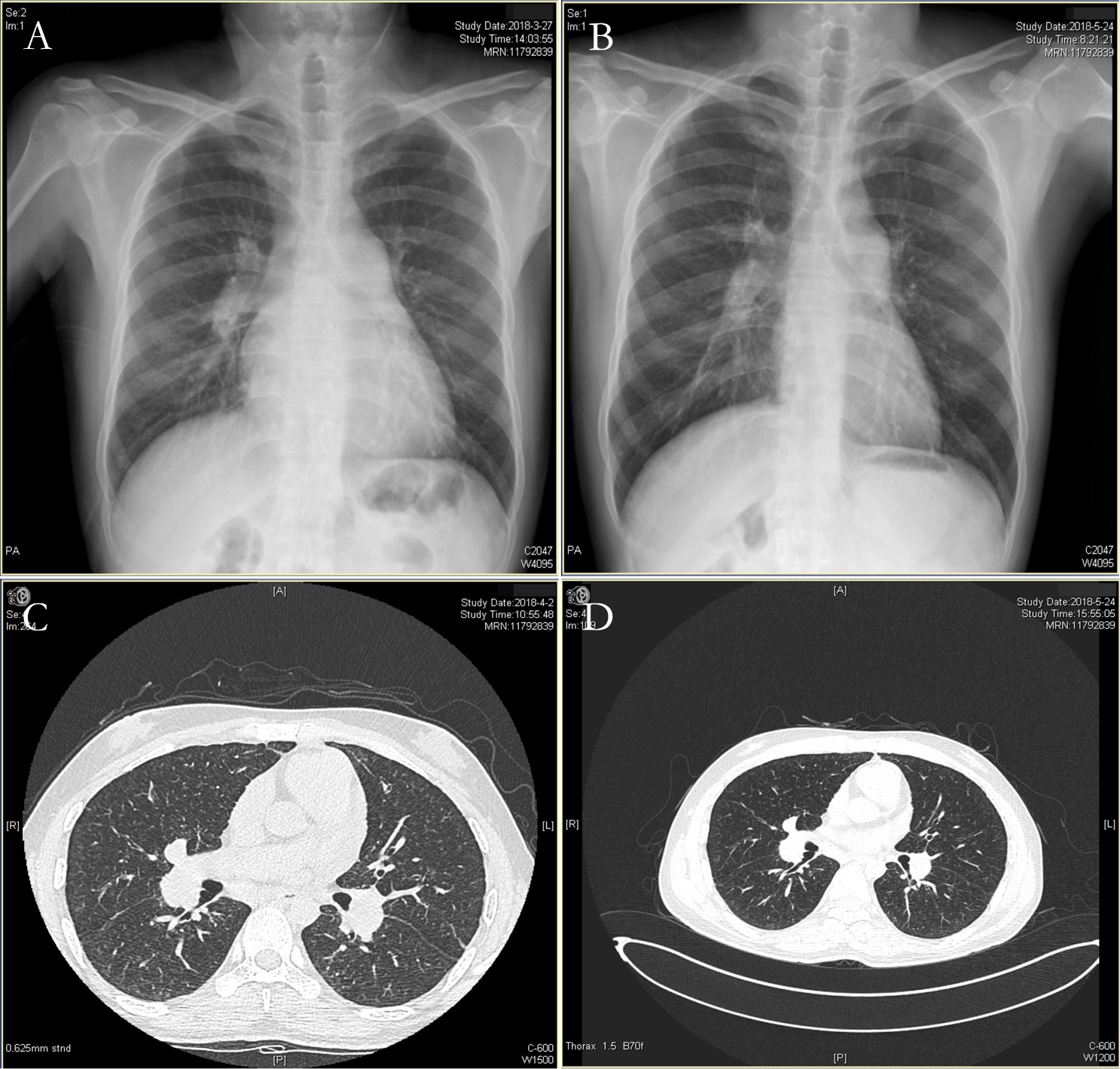
Additional file 1: **Figure S1** Chest X-ray and computed tomography pre- (A, C) and post- (B, D) PAH targeted therapy in Patient 3
